# Circular RNA hsa_circ_0006091 as a novel biomarker for hepatocellular carcinoma

**DOI:** 10.1080/21655979.2021.2006952

**Published:** 2022-02-03

**Authors:** Yongwei Zhang, Jun Li, Quanwei Cui, Panyi Hu, Shuangjiu Hu, Yeben Qian

**Affiliations:** a Department of Gastrointestinal Surgery, Anqing First People’s Hospital Affiliated to Anhui Medical University, Anhui, Anqing, China; b Department of General Surgery, First Affiliated Hospital of Anhui Medical University, Hefei, China

**Keywords:** Hepatocellular carcinoma, hsa_circ_0006091, diagnostic markers, biological information

## Abstract

Circular RNAs (circRNAs) are stable and extensively distributed non-coding RNA molecules that are differentially expressed in liver cancer tissues in the human body. In this study, we aimed to investigate circRNA as a novel candidate biomarker for hepatocellular carcinoma (HCC). For three groups of HCC and neighboring healthy tissues, the differentially expressed circRNAs were identified through high-throughput sequencing analysis. Reverse transcription PCR (RT-PCR) and quantitative polymerase chain reaction (qPCR) were employed for the evaluation of circRNAs that show an elevated expression level in HCC. The obtained results revealed the significantly differential expression of hsa_circ_0006091 in HCC. Then we obtained their target genes through biological analysis, followed by verifying the underlined target genes, and the regulator of G-protein signaling 12 (RGS12) showed an elevated expression level in HCC tissues. Finally, receiver operating characteristic (ROC) curve analysis was conducted on AFP, RGS12, and hsa_circ_0006091, and combined analysis was performed. Furthermore, hsa_circ_0006091 is a novel candidate biomarker for HCC and could improve the diagnostic strategies, prediction, and follow-up of HCC patients. The joint diagnosis of the hsa_circ_0006091&AFP and hsa_circ_0006091&RGS12 has diagnostic significance and can be used as a molecular marker for HCC diagnosis.

**Abbreviations:** AUC:area under the ROC curve; ROC:Receptor Operating Characteristics; bp:base pair;mRNA:Messenger Ribonucleic acid;ceRNA:Competing endogenous RNA; RT-qPCR: Real time-quantitativen PCR technology; circRNA: circular RNA; HCC:Hepatocellular carcinoma;miRNA:microRNA;KEGG:Kyoto Encyclopedia of Genes and Genomes; RGS12:regulator of G-protein signaling 12; AFP:alpha fetoprotein; ncRNAs:non-coding RNAs; GEO:Gene Expression Omnibus; FDR:false discovery rate

## 1. Introduction

Despite being the sixth most prevalent cancer diagnosed in the world, liver cancer appears to be the fourth major cause of cancer deaths worldwide due to its poor prognosis [1,2]. Males have a two to three times higher morbidity and mortality rate than females, therefore, liver cancer is the fifth most common cancer worldwide and the second leading cause of death in men [3]. Despite advances in targeted therapies, diagnosis, immunotherapy, and surgical techniques, the 5-year overall survival rate in HCC patients are still not optimal [4]. The main reasons are the low rate of diagnosis in the initial stages, recurrence after surgery, and distant metastasis [5]. AFP is a significant biomarker that helps in the detection of liver carcinoma, although diagnostic approaches based on AFP are still far from satisfactory due to its poor sensitivity and specificity [6,7]. Therefore, there is an urgent need to explore candidate molecular markers for the HCC detection that will improve the diagnostic strategies and help in treating liver carcinoma.

CircRNAs are produced by alternative splicing of a special pre-mRNA (pre-mRNA) during the transcription process [8]. They belong to non-coding RNAs (ncRNAs), which is a new class without a 5’-end cap or 3’-end poly (A) tail [9]. Moreover, circRNAs are mostly found in the cytoplasm and are remarkably stable [10]. They can interact with microRNAs (miRNAs) and prevent the functional expression of miRNA and its target genes, thereby exerts their biological effects [11]. CircRNA may provide resistance to exonuclease-facilitated digestion, and is more conservative and stable than the corresponding linear components, and has a longer half-life than linear RNA due to the endogenous transcript with a covalently closed loop [12]. Due to these features, circRNA can lead as a potential biomarker. Currently, circRNA has been identified to play a key regulatory role in various pathological or physiological processes, including reproduction [13], cardiovascular system [14], metabolism [15], psychiatric disease [16], and tumorigenesis [17,18]. The abnormal expression profile of circRNA is potentially utilized for the characterization of HCC at the molecular level, and some circular RNA biomarkers of such nature have been extensively identified in various studies [19,20], that significantly contribute to elucidating the underlying molecular mechanisms involved in the HCC incidence. However, until now, the circRNA biomarkers used to predict HCC in clinical practice have rarely been discovered. Therefore, finding new and specific circRNA biomarkers to study and track the progression and prognosis of HCC will be critical.

In this study, the circRNA expression profiles were assessed in 3 pairs of HCC and corresponding neighboring tissues via high-throughput RNA sequencing. The sequencing data were analyzed and screened, and four circRNAs having high expression levels were chosen. Then fluorescence-based qRT-PCR was employed for verifying the sequencing data. The expression of hsa_circ_0006091 in HCC tissues and surrounding tissues was shown to be significantly different. Furthermore, the expression level of hsa_circ_0006091 was confirmed in 52 pairs of HCC and corresponding adjacent tissues and 11 pairs of normal liver tissues, and the relationship between the expression level of hsa_circ-0006091 and individual clinicopathological parameters of HCC patients was investigated. Meanwhile, the prediction of downstream miRNA and targeted genes was performed and the circRNA-miRNA-mRNA regulatory network was constructed for the evaluation of the crucial biological functions of circ-0006091. GO and KEGG analyses were carried out to discover the role of target genes. According to the competitive endogenous RNA (ceRNA) theory, an increase of non‑coding RNAs (ncRNAs) level can adsorb miRNA and reduce the inhibition on the target genes, leading to an increase in these genes expression [21,22]. In addition, some studies have shown that circRNA can regulate the expression of its parental genes by isolating miRNA, implying that their parental genes can potentially become its downstream target genes [12,23–25]. So, theoretically, hsa_circ_0006091 should be positively correlated with the expression level of its target mRNAs or parental gene. Hence, we hypothesize whether the target mRNAs or parental gene of hsa_circ_0006091 can also be used as a molecular marker. Therefore, we conducted qPCR to verify its potential target mRNA, including its parental gene RGS12. The obtained results indicated that RGS12 has a high expression level in HCC tissues than matched normal tissues. We then performed a joint analysis of the ROC curve.

This study aims to find more sensitive and specific molecular markers for HCC. The target circRNA was screened by sequencing and was then validated through further experimental data. Finally, the circRNA that fir the criteria was found to improve the rate of liver cancer diagnosis and reduce the rate of liver cancer death.

## 2. Methods

### 2.1. Patient samples

All the samples used in this study were collected from HCC patients after surgery in the First Affiliated Hospital of Anhui Medical University, from January 2018 to August 2020. The samples consisted of tumor tissues and matched healthy tissues (n = 52) of confirmed HCC patients. Simultaneously, samples from benign liver disease patients (n = 7 as hepatic hemangioma; 5 as a hepatic cyst) and the pre- and postoperative plasma and serum of HCC patients were also included.

The total patients included in the study were declared as confirmed primary cases through clinical and histological examinations. The specimens were stored in the refrigerator at −80°C until further use. Before surgery, the HCC patients did not receive any of the chemotherapy, radiotherapy, or targeted therapy. To avoid the interference of chronic diseases such as diabetes, HCC patients with chronic diseases were excluded from this study. The clinical features of HCC and benign liver disease are shown in Supplementary Tables S1–2, respectively. All patients had given their informed consent before surgery, which informed them that their samples would be used for biomedical research. The study was approved by the Ethics Committee of the First Affiliated Hospital of Anhui Medical University (pj-2020-12-14).

### 2.2. High throughput sequencing

The RNA sequencing experiment process included circRNA extraction, circRNA sample quality detection, library construction, library purification, library detection, library quantification, sequencing cluster generation, and computer sequencing, as depicted in Figure S1. Part of the process was carried out by Genewiz Co., Ltd. (Suzhou, China). We executed strict quality control on each step of the experimental process to confirm the precision and reliability of the data source. Post qualification of the test, the different libraries were mixed according to the requirements of effective concentration and target offline data volume and then sequenced on the Illumina platform. Genes were identified for up or down regulation in comparison with the control by using Log 2-fold change in the FPKM value of each gene in all samples. Under the accession number GSE159220, the RNA-Seq data were uploaded to the Gene Expression Omnibus (GEO) database. The relevant information of the 3 patients used for sequencing is listed in Table 1.

**Table 1.** The clinicopathological parameters of the three pairs HCC tissues used for the analysis of circular RNAs expression profiles in high-throughput sequencing

Patient No.	Age	Gender	Tumor size	TD	LNM	DM	TNM stage	HBV
1	45	Male	5.2	Moderate	NO	NO	T2N0M0	Positive
2	72	Male	6.3	Moderate	NO	NO	T2N0M0	Positive
3	48	Male	3.9	Poorly	yes	NO	T3N1M0	Negative

TD,Tumor differentiation; LNM,LN metastasis;DM, Distant metastasis.

### 2.3. RNA extraction and qRT-PCR

Using the TRIzol reagent (Invitrogen), the total RNA was extracted from the target tissues following instructions from the manufacturer. For circRNAs, RNase R was used to degrade linear RNAs, and amplified by a divergent primer. Certain divergent primers that spanning the back-splice junction site of circRNAs were also designed. All the primers used in the study were synthesized by Sangon Co., Ltd (Shanghai, China). To quantify the amount of circRNA and mRNA, the complementary DNA (cDNA) was synthesized with the help of the M-MLV Reverse Transcriptase (Invitrogen). Using the SYBR Premix Ex Taq (TaKaRa) and Prime Script RT Reagent Kit (TaKaRa), the qRT-PCR analysis was performed for circRNA and mRNA. Moreover, β-actin was utilized as an internal control, and all procedures were repeated three times. To represent the relative gene expression levels, the fold change (2^−ΔΔCt^) was used. Table S3 displays all primer sequences for each mRNA and circRNA used in this study.

### 2.4. Ranger sequencing

Due to the short product fragment of circ0006091 (123bp), its sequence cannot be identified by ordinary Sanger sequencing. So, competent cells were cultured via the T vector transfection method and then sequenced. In the first step, we cut the gels of the target product and then placed it into an eppendorf tube (1.5 ml), then membrane binding solution (1ul) was added and place in a 65°C water bath until it was completely dissolved. After that, 650 µl of membrane washing solution was added, followed by centrifugation and discarding the filtrate. Bacterial transformation, and competent cell assembly were conducted after going through the steps of the T vector (Sangon Biotech) cloning. The product was sent to Generalbiol Co., Ltd. (Shanghai, China) for Sanger sequencing.

### 2.5. qRT-PCT for detecting plasma circRNA

First glycogen (Invitrogen) and TRIzol reagent (Invitrogen) were added to the serum, then 10% sodium acetate (Invitrogen) and pre-cooled isopropanol (equal in volume) were introduced to the extracted RNA supernatant. The RNA was then precipitated for 7–8 hours at −80°C overnight, with the goal of depositing low molecular weight RNA in the mixed solution. In the next step, cDNA was synthesized with the help of M-MLV reverse transcriptase (Invitrogen). qPCR was performed via SYBR Premix Ex Taq (TaKaRa), and specific data were obtained by using the LightCycler 96 instrument (Roche). Finally, the results were analyzed according to the previous qPCR results in the tissue.

### 2.6. Bioinformatics analysis

CircInteractome (https://circinteractome.nia.nih.gov) and circBank (http://www.circbank.cn/index.html) were used for predicting candidate miRNAs of hsa_circ_0006091. Venny 2.0 (http://bioinfogp.cnb.csic.es/tools/venny/index.html) was used to draw the Venn diagram of miRNA for observing the intersection of the two databases. Then the MiRWalk (http://mirwalk.umm.uni-heidelberg.de) and TargetScan (http://www.targetscan.org) were used for the prediction of the downstream target genes of miRNA. With the help of the GDCRNATools Package, the circRNA-miRNA-mRNA network was constructed, and its visualization was carried out with Cytoscape software (version 3.7.1). Furthermore, for an extensive understanding of the functions of ceRNA and more accurately screening the downstream target genes, the GO and KEGG pathways of these genes were analyzed by bubble mapping of the R package GGplot2.

### 2.7. Statistical analysis

The data were analyzed with the help of GraphPad v7.0, SPSS v23.0, and R software 3.6.1. Quantitative data were summarized and expressed as mean ± standard error of the mean (SEM). The adjusted t-test was utilized for the analysis of data related to independent groups, and the paired t-test was performed to assess the difference in expression of circRNA in HCC tissues and matching adjacent tissues. Chi-square test analysis was used to evaluate the correlation between the expression level of circRNA and the clinicopathological parameters of HCC. With the help of AUC (area under the ROC curve) and ROC (Receptor Operating Characteristics), the circRNA specific diagnostic value was evaluated. P-value<0.05 was considered statistically significant.

## 3. Results

In this study, differentially expressed circRNAs were obtained using high-throughput sequencing. The high expression of circRNA was validated. The bioinformatics analysis was performed to screen and confirm the downstream target genes. Finally, ROC curve analysis and combined analysis were performed on target circRNA, AFP, and target genes respectively. Identification of more sensitive and specific molecular markers for the diagnosis of HCC can be expected.

### 3.1. Identification of a potential hsa_circRNA marker for HCC

**Figure 1.**
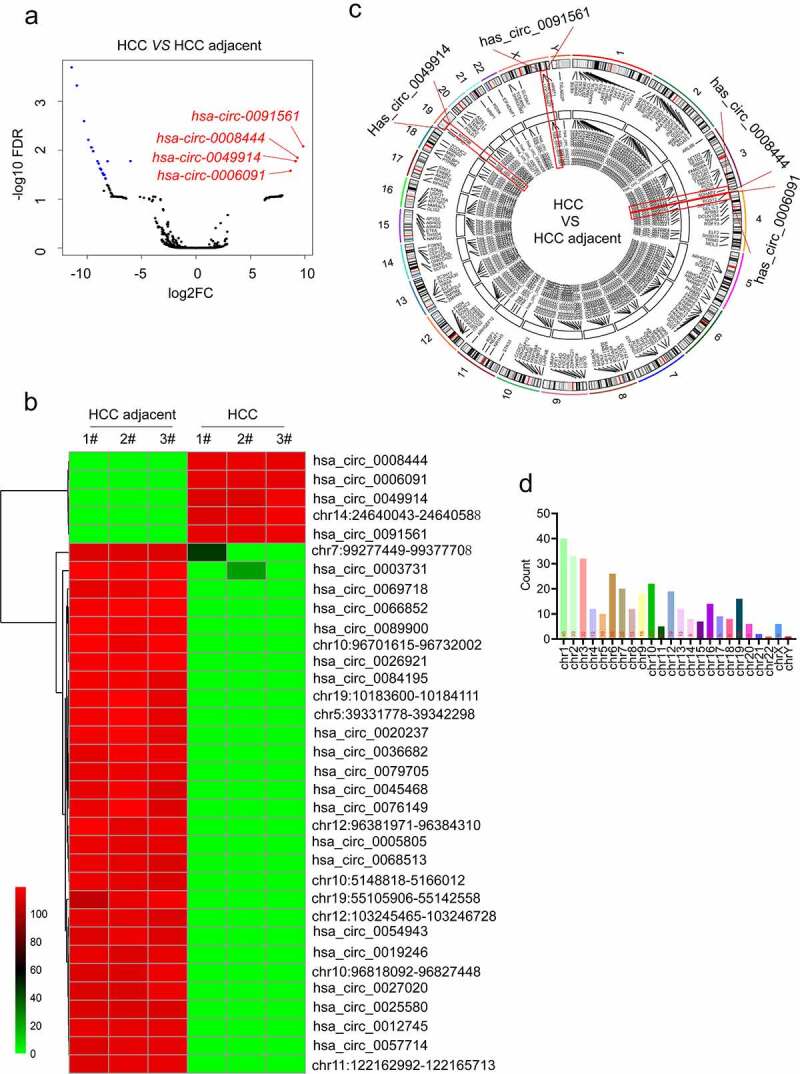
The identification of a potential circ_RNA marker for HCC. (a) DE-circRNAs volcano map, significant De-circRNA red points indicate up-regulation, blue points indicate down-regulation; the x-axis indicates the fold change of circRNA expression in various samples; the y-axis represents the statistical significance of the difference in circRNA expression. (b) DE-circRNA data were adjusted the P-value and obtained 5 up-regulated and 29 down-regulated chromosome fragments, some of which could not be identified by the circbank and circbase databases, and finally, we got 4 up-regulated and 19 down-regulated expressed circRNAs. (c) the name, hosting gene, position on the chromosome, and average expression of each DE-circRNAs were displayed by the Circos diagram. In this study, the four genes have been marked by the red box. (d). The statistical graph of the number of differential circRNA corresponding to each chromosome is described.

In this study, we analyzed the circRNA expression profile of HCC tissue samples via sequencing for the identification of a significant diagnostic marker for HCC. Herein, we also revealed some new circRNAs that had not been reported before. Genes with |fold change| ≥ 2 and false discovery rate (FDR) < 0.05 were considered differentially expressed. Taken together, a total of 339 different circRNAs were obtained. The volcano map was constructed, followed by marking the top 4 highly expressed circRNAs as shown in Figure 1(a). We further sorted out the differential circRNA data, adjusted the P-value, and obtained 5 up-regulated and 29 down-regulated chromosome fragments. Among these chromosomal fragments, some of them could not be identified by circbank and circbase databases. Hereafter, we obtained 4 up-regulated, and 19 down-regulated expressed circRNAs (Table S4). The heat map was constructed using the heatmap package of R language (Figure 1(b)). The Circos diagram was constructed to illustrate the name, hosting gene, position on the chromosome, and average expression of each different circRNA in a more intuitive way (Figure 1(c)). To make the picture more clearly visible, some circRNAs that were overlapped with parental genes were removed [26]. The statistical graphs of several differentially expressed circRNA(DE-circRNAs) corresponding to each chromosome are presented in Figure 1(d). It can be seen that the numbers of DE-circRNAs on chromosomes 1, 2, and 3 are the largest. After analysis, the four most up-regulated circRNAs such as hsa_circ_0008444, hsa_circ_0006091, hsa_circ_0049914, and hsa_circ_0091561 were obtained. The specific Log2FC and p-value of the four circRNAs in the underlined sequencing data are described as follows: hsa_circ_0091561 (log2FC = 9.32, p = 0.000137822) located at chrX:130928351 to 130,959,393 with spliced length 876 bp and produced by LOC286467. hsa_circ_0008444 (log2FC = 9.15, p = 0.000194432) located at chr3:195,415,403 to 195,426,078 with spliced length 10675bp, and produced by SDHAP2. hsa_circ_0049914 (log2FC = 9.14, p = 0.000217995) located at chr19:17,212,469 to 17,313,692 with spliced length 4936bp, and produced by MYO9B. hsa_circ_0006091 (log2FC = 8.67, p = 0.000613286) located at chr4:3,317,796 to 3,344,780 with spliced length 2099bp, and produced by RGS12.

### 3.2. Hsa_circ_0006091 up-regulation in HCC samples.

**Figure 2.**
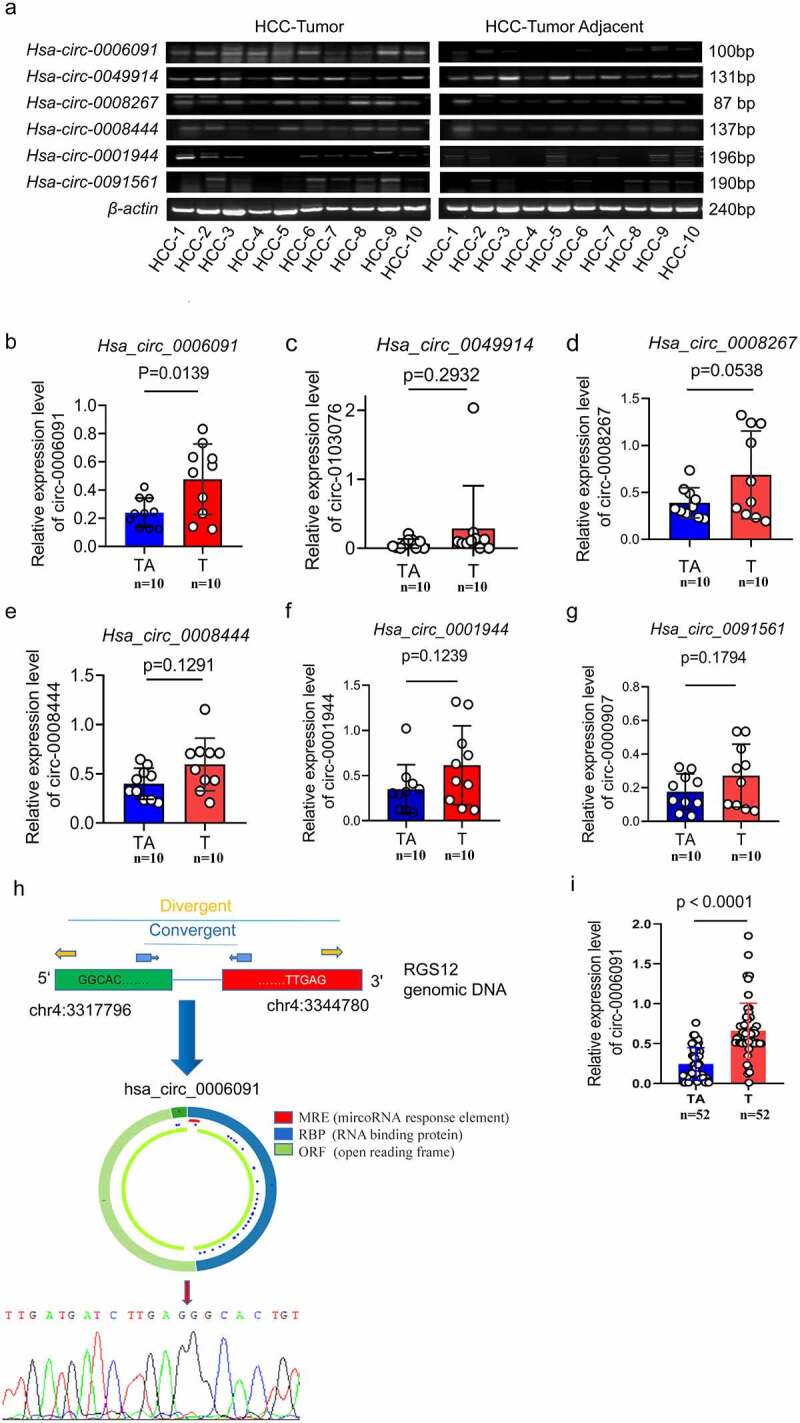
Hsa_circ_0006091 was considerably upregulated in HCC tissues. (a) The RT-PCR was employed to evaluate the expression of four candidate genes (hsa_circ_0006091, hsa_circ_0008444, hsa_circ_0049914, hsa_circ_0091561) in HCC tumor tissues (n = 10) and matched adjacent normal tissues (n = 10). Each band indicates a diverse patient sample. (b-e) hsa_circ_0006091, hsa_circ_0008444, hsa_circ_0049914 and hsa_circ_0091561 levels were determined through q-PCR in HCC tissues (n = 10) compared with paratumor tissue samples (n = 10), respectively. (f) The schematic diagram displaying that hsa_circ_0006091 was derived from RGS12. The head-to-tail splicing junction sequence was identified via the Sanger sequence. The structural diagram of hsa_circ_0006091 is from CSCD. T, tumor. TA, tumor adjacent tissue. (i) Further verification of hsa_circ_0006091 in 52 pairs of HCC and adjacent tissues.

To further determine the sensitivity and specificity of these four circRNAs in HCC, we evaluated 10 pairs of HCC tissues and matched adjacent normal tissues by the RT-PCR method. At the same time, we selected two other circRNAs, hsa_circ_0001944 and hsa_circ_0091561, that are highly expressed in sequencing but have not been studied for verification. The related primers are presented in supplementary Tables S2. In HCC tissues the positive expression rate of hsa_circ_0006091(80%,8/10) was found to be higher as compared with the adjacent tissues (10%,1/10) as depicted in Figure 2(a). Subsequently, we assessed the relative expression level of these circRNAs via qPCR analysis (Figure 2(b-g)). Only hsa_circ_0006091 in HCC tissues showed an elevated level of expression than that in paracancerous tissues with significant differences (p < 0.05, paired t-test). Next, hsa_circ_0006091 was taken as the main target of our further research. To eliminate the interference of cognate linear mRNA with a similar structure, and to further determine whether it is a circular structure or not, we performed Sanger sequencing on the product of gels. According to the results of Sanger sequencing, hsa_circ_0006091 exist in a circular structure, as depicted in (Figure 2(h)). We performed qPCR on a total of 52 cases of HCC and adjacent tissues to further verify hsa_circ_0006091 and found significant differences (Figure 2(i)). In addition, the correlation analysis of clinical parameters found that the level of hsa_circ_0006091 expression was significantly related to the tumor size and TNM Stage, with P values of 0.002 and 0.01, respectively (Table 2).

**Table 2.** Expression of circ0006091 and clinical features of LUSC patients (n = 52)

Variables	Total number	Circ0006091 expression		P value	χ2
		High (n = 26)	Low (n = 26)		
Ages (years)				0.575	0.315
<60	30	16	14		
≥60	22	10	12		
Gender				0.126	2.342
Male	37	21	16		
Female	15	5	10		
Tumor size				0.002*	9.321
<5	25	18	7		
≥5	27	8	19		
Differentiation				3.250	0.071
Well or moderately	36	15	21		
Poorly	16	11	5		
Vascular invasion				1.564	0.211
No	38	17	21		
Yes	14	9	5		
Distant metastasis				1.415	0.2344
No	49	23	26		
Yes	3	3	0		
TNM Stage				0.010*	6.718
I, II	33	12	21		
III, IV	19	14	5		
AFP (ng\ml)				0.416	0.663
<10	19	12	7		
≥10	33	17	16		
Hepatitis virus status				0.150	2.073
HBsAg (+)	33	19	14		
HBsAg (-)	19	7	12		

(a) The mean ± standard deviation is used to represent a continuous variable with a normal distribution. The quartile range is used to indicate a skewed distribution. The median expression value of hsa_circ_0006091 is taken as cutoff. In each 26 patients, low expression of hsa_circ_0006091 was characterized as a value below the 50th percentile. (b) χ2 test, *P < 0.05. (c) TNM stage: T, Tumor; N, nodes; M, Metastasis. (d) AFP, alpha-fetoprotein; HBsAg(+), hepatitis B surface antigen (+) means HBV infection, conversely, HBsAg(-) indicates uninfected by HBV.

### 3.3. circ-0006091-miRNA-mRNA network pathway and prediction of downstream genes

**Figure 3.**
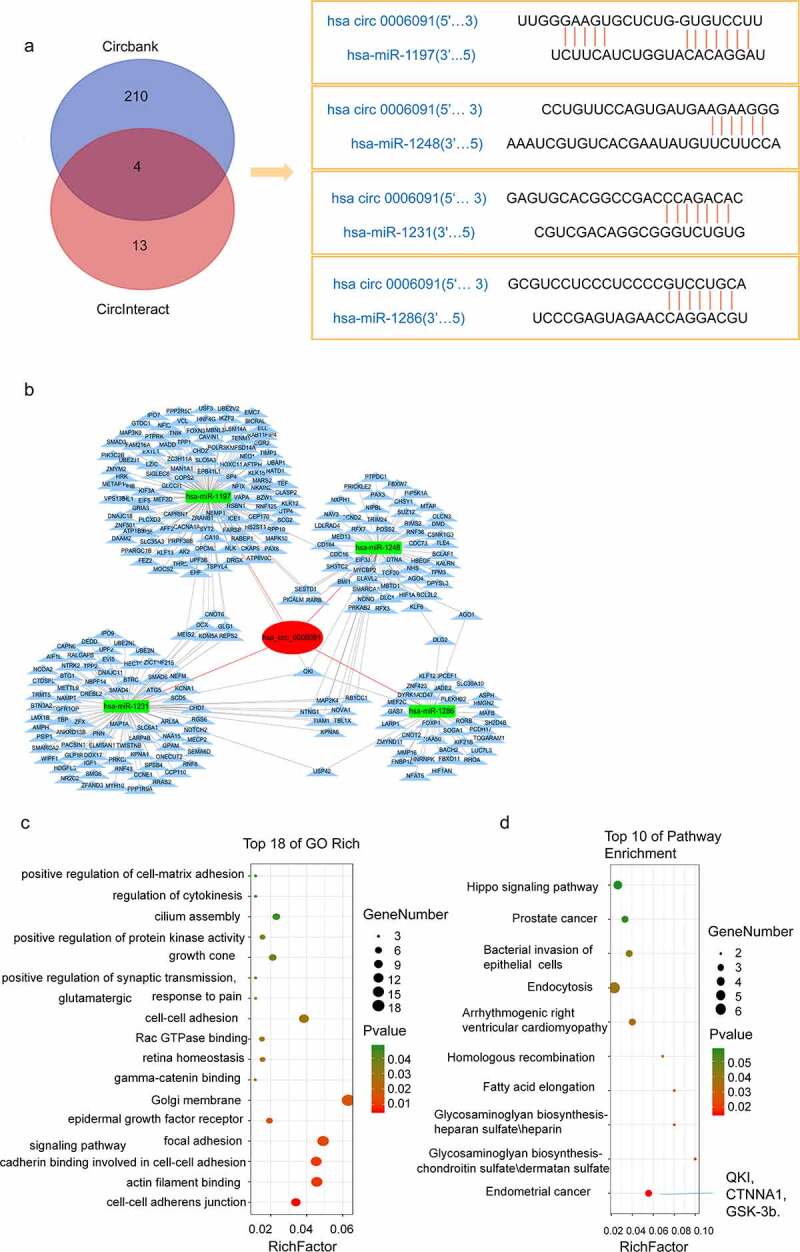
The circ-0006091-miRNA-mRNA network pathway and prediction of downstream genes. (a) Predicted the potential miRNAs related to hsa_circ_0006091 through the circBank and circinteractome databases, and used the Venn diagram for the intersection. Finally, 4 miRNAs with an intersection were obtained. Then, circinteractome was employed for the prediction of the circRNA‑miRNA pairing sites between hsa_circ_0006091 and the miRNA *i.e*., hsa-miR-1197, hsa-miR-1248, hsa-miR-1231, and hsa-miR-1286. (b) The network diagram of hsa_circ_0006091, which has four miRNAs and their downstream mRNAs. (c) GO analysis indicates that target genes associated with candidate circRNAs contribute to some key biological functions. The abscissa represents the richfactor, the ordinate indicates the enriched pathways. (d) KEGG signaling analysis was used to confirm the role of the potential target genes. The abscissa means the richfactor of genes and the ordinate strands for the enrichment pathway. GO, gene ontology; KEGG, Kyoto encyclopedia of genes and genomes.

The circRNA expression level was found to be positively correlated with the expression of the target mRNA. To reveal whether the target mRNA downstream of hsa_circ_0006091 is also differentially expressed for working as a diagnostic marker for HCC, a network pathway of hsa_circ_0006091 was constructed for examining the downstream target genes in a precise manner. First, CircInteractome and circBank were selected to predict the miRNA downstream of hsa_circ_0006091, and the intersection was taken to obtain four intersection miRNAs, such as hsa-miR-1197, hsa-miR-1248, hsa-miR-1231, and hsa-miR-1286. TargetScan showed the binding sites of hsa_circ_0006091 as shown in Figure 3(a). Next, we used MiRWalk and TargetScan databases to predict the downstream target genes of these miRNAs and constructed a co-expression network of circRNA-miRNA-mRNA using Cytoscape (Figure 3(b)).

Herein, we used KEGG and GO pathway analyses for further exploring the functions of the identified targeted genes. GO analysis revealed that target genes associated with circRNAs candidates are frequently involved in certain biologically important functions (Figure 3(c)). However, KEGG pathway analysis identified that candidate mRNAs are considerably linked with various key pathways, including endometrial cancer, fatty acid elongation, and homologous recombination pathways (Figure 3(d)). Therefore, these functional analyses have identified significantly relevant pathways that are important in the development of HCC. In addition, we can see that the endometrial cancer pathway directly related to tumor development contains three genes of QKI, CTNNA1, GSK-3b.

### 3.4. Overexpression of circ-0006091 target genes in HCC

**Figure 4.**
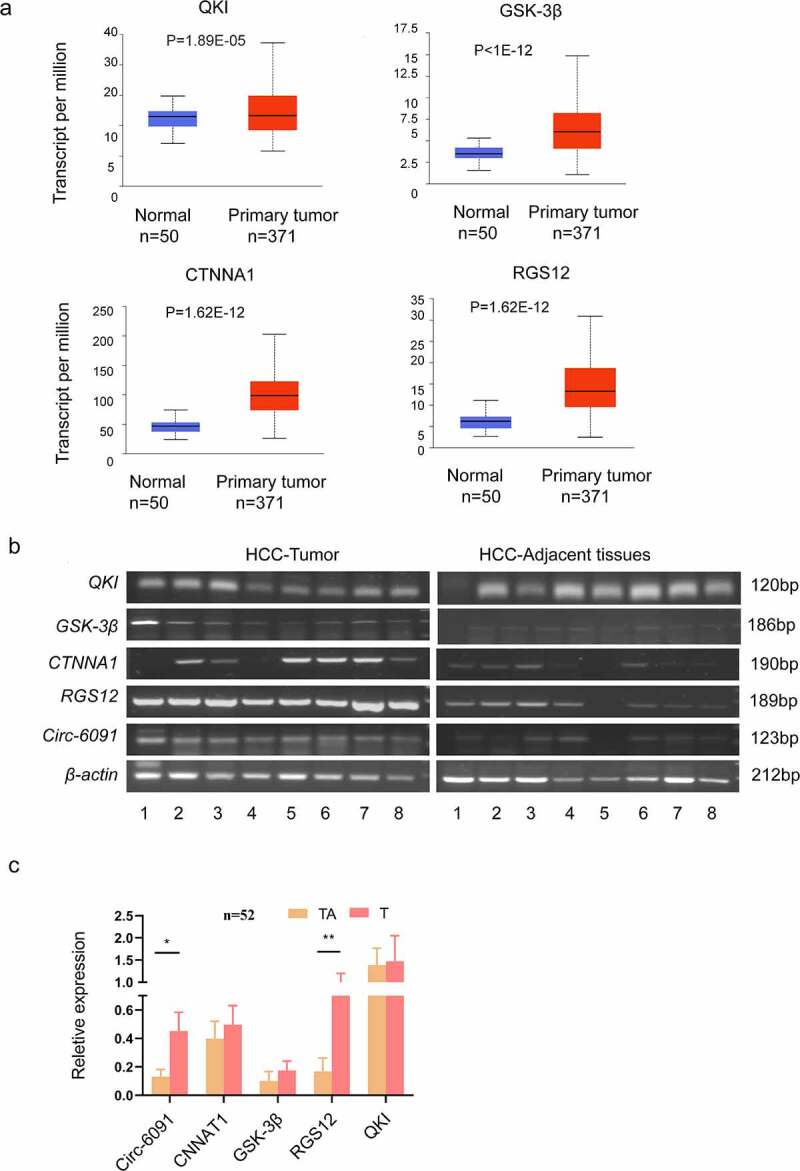
The elevated expression level of circ-0006091 target genes in HCC. (a) the histogram of QKI, CTNNA1, GSK-3b, and RGS12 was obtained through Ualcan, respectively. (b) The expression of the genes *i.e*., CTNNA1, QKI, GSK-3b, and RGS12 were evaluated via RT-PCR in HCC tumor tissues (n = 8) and matched normal tissues (n = 8). Each band means a different patient sample. (c) The expression of these genes (CTNNA1, QKI, GSK-3b, RGS12, and hsa_circ_0006091) were determined by q-PCR in HCC tumor tissues (n = 52), and matched normal tissues (n = 52).

We used GO and KEGG analysis to analyze many cancer-related genes, including QKI, CTNNA1, GSK-3b, and RGS12, which had not been previously identified in HCC. At the same time, the histogram of these genes was obtained through the Ualcan database (http://ualcan.path.uab.edu/cgi-bin/ualcan-res.pl) (Figure 4(a)). In this view, we selected QKI, CTNNA1, RGS12, and GSK-3b, and hsa_circ_0006091 for RT-PCR (Figure 4(b)) and qRT-PCR evaluation (Figure 4(c)). Based on these experiments, RGS12 was found to be up-regulated in HCC tissues, in comparison with neighboring tissues. There was a significantly different expression.

### 3.5. Monitoring the prognosis and recurrence of HCC patients through the plasma level of hsa_circ_0006091.

**Figure 5.**
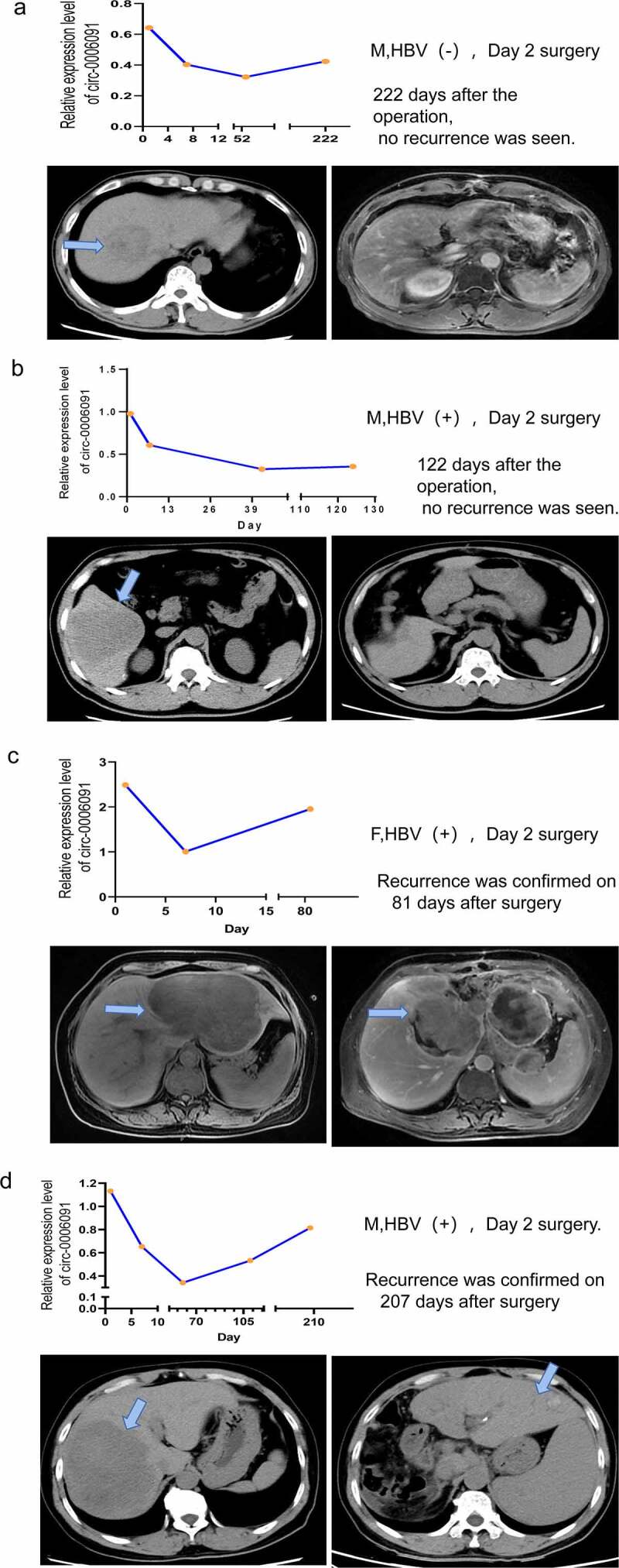
The monitoring of the prognosis and recurrence of HCC patients through the plasma level of hsa_circ_0006091. (a-d) The above panel respectively represents the expression level of hsa_circ_0006091 on the first day before the operation, the 7th day after the operation, and the subsequent follow-up. The below panel is the abdominal CTs of four HCC patients before and after surgery. The green arrow indicates the location of the tumor on the CT. Among them, the patients in Figure 5(a,b) showed no obvious signs of recurrence after the postoperative review. The patients in Figure 5(c,d) showed tumor recurrence after reexamination.

The patient’s blood was collected 1 day before surgery and 7 days after surgery, with long-term follow-up to understand the expression of hsa_circ_0006091 in the same HCC patient at different times. A total of 13 patients diagnosed with HCC were recruited in the study, and 1 postoperative pathological CCA patient and some patients who were not admitted to the hospital were discharged. Taken together, 4 patient’s return visit records were obtained and analyzed. Long-term blood monitoring was performed on HCC patients to examine the recurrence of the tumor and the relationship between serial alterations in hsa_circ_0006091 levels. The changes in plasma circRNA levels before and after surgery in 4 HCC patients were observed. The serum of an HCC patient with HBV (-) was used to monitor the hsa-circ-0006091 level one day before surgery, and 7 days, 52 days, and 222 days post-surgery. The symptoms of tumor recurrence were not observed in the underlined patient on CT scan 222 days post-surgery, as depicted in Figure 5(a). The serum hsa_circ_0006091 level of an HBV-positive patient was tested on one day before surgery, 7 days after surgery, 43 days, and 122 days (four months). The patient’s CT scan after four months of surgery showed no obvious signs of tumor recurrence (Figure 5(b)). An HBV-positive patient was monitored for the serum hsa_circ_0006091 level on the first day before the operation, the 7th day after the operation, and the 81st day. The patient’s CT recurrence was found on the 81st day after the operation as depicted in Figure 5(c). An HBV-positive patient monitored serum hsa_circ_0006091 levels on the day before surgery, day 7, day 65, and day 207 respectively. In the patient’s CT scan report, as shown in Figure 5(d), tumor recurrence was detected 207 days after surgery. We can see from the statistics in Figure 5(d) that the level of hsa-circ-0006091 in patients after the operation decreased and then increased. This coincides with the time of tumor resection and postoperative recurrence. In summary, these data indicate that hsa_circ_0006091 can be used as a new biomarker for monitoring HCC recurrence.

### 3.6. The combination of circ-0006091 and RGS12/AFP improves diagnostic performance for HCC.

**Figure 6.**
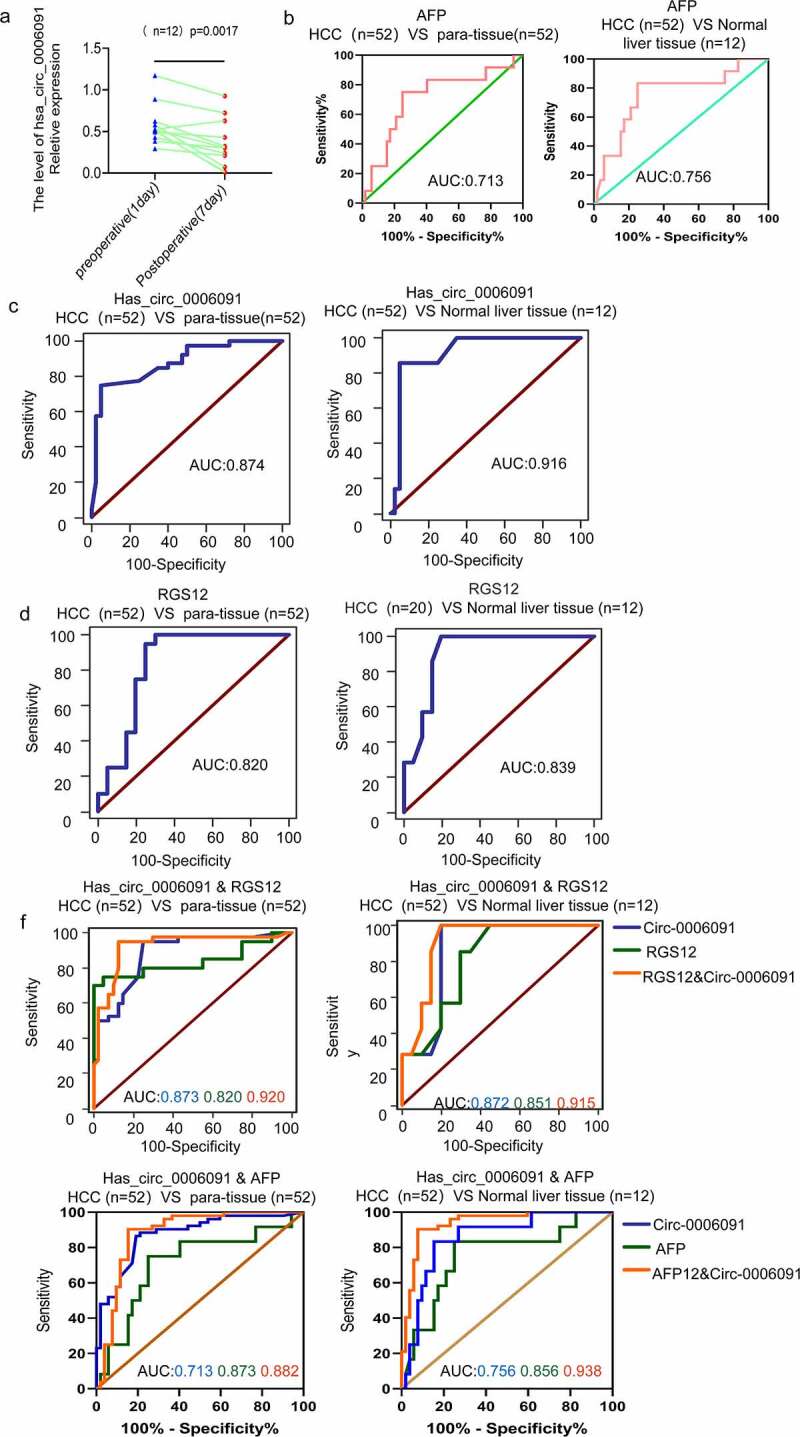
The combination of hsa_circ_0006091 and AFP, hsa_circ_0006091 and RGS12 enhances the diagnostic strategies for HCC. (a) Serum levels of hsa_circ_0006091 were measured in 12 patients 1 day before and 7 days post-surgery. (b-d) ROC curve analysis of AFP, hsa_circ_0006091 and RGS12 differentiated HCC tissues and between paracancer and benign liver tissues, respectively. (f) ROC curve was used for joint diagnosis of hsa_circ_0006091&AFP and hsa_circ_0006091&RGS12, respectively in HCC s (n = 52) versus adjacent tissues (n = 52), HCC versus benign liver disease tissues (n = 12).

Serum levels of hsa_circ_0006091 were measured in 12 patients one day before and 7 days after surgery, which showed that the levels of hsa_circ_0006091 have a significant decline after HCC surgery as depicted in Figure 6(a). To examine the role of hsa_circ_0006091 as a potential HCC biomarker and to precisely monitor the disease, the accuracy of the hsa_circ_0006091 diagnosis was evaluated using ROC curve analysis. We also measured the optimal cutoff value of the hsa_circ_0006091 expression threshold along with the Youden index (sensitivity + specificity −1), and maximum sensitivity and specificity. Comparing the HCC group with the adjacent tissues, the area under the curve of ROC (AUC) was found to be 0.874 [95% confidence interval (CI) = 0.794–0.931; p < 0.0001]. The sensitivity and specificity of hsa_circ_0006091 for the diagnosis of HCC were found to be 0.865 and 0. 808, respectively. The AUC was found to be 0.916 in comparison with the healthy control group as depicted in Figure 6(c). The levels of AFP and RGS12 expression and the area under the curve were compared between the HCC group and the adjacent normal tissues. ROC was also compared with that of the healthy control group (Figure 6(b,d), and Table S4 for detailed ROC-related information). Taken together, the combined RGS12 or AFP diagnosis was found to be more sensitive in comparison with hsa_circ_0006091 individually. ROC curve was used for joint diagnosis of hsa_circ_0006091 and AFP/RGS12, respectively in HCC s (n = 52) versus adjacent tissues s (n = 52), HCC versus benign liver disease tissues (n = 12) (Figure 6(f)). The results revealed that the combined diagnosis of hsa_circ_0006091 and AFP/RGS12 was more sensitive and specific.

## 4. Discussion

Liver cancer is one of the highly predominant malignant tumors across the globe. Because the tumor is prone to return after surgery and the patients are typically asymptomatic or have moderate symptoms in the early stages, the mortality rate from liver cancer remains high. Although the current diagnostic level and methods for liver cancer are continually evolving, the diagnostic strategy and prognosis remain unsatisfactory. Therefore, candidate biomarkers are required to improve diagnostic strategies in the early stages of HCC as well as to assess its prognosis rates. AFP is the most commonly used biomarker for the detection of HCC in clinical practice, but several studies have reported that in around 40% of HCC patients, the AFP expression level is normal, which indicates its low level of sensitivity [12,27]. So, there is an urgent need to explore potential and sensitive biomarkers. Compared with linear RNAs, circRNAs have a special covalently closed circular structure, which makes them more resistant to exonucleases. CircRNA is abundant, conserved, and tissue-specific and due to these features, it can play a significant role in several diseases [28]. Many studies have confirmed that circRNA significantly contributes to the occurrence and growth of many tumors.

It is differentially expressed in many tumors and can be used as a potential biomarker of the tumor. David et al. found that hsa_circ_0001445 can serve as a novel biomarker of stable coronary artery disease (SCAD) [29]. Guo et al. revealed that circ_0006156 in papillary thyroid cancer might serve as a novel biomarker [30]. Tian et al. reported that circ_0044516 can work as a candidate indicator for prostate cancer [31]. Luo et al. revealed that the hsa_circ_0000190 level might act as a significant biomarker to monitor the disease status of lung cancer [32]. Xiao et al. identified that circFADS2 serves as an oncogenic biomarker in colorectal cancer [33]. Liang et al. revealed that circCDYL can serve as a key predictive and prognostic molecule for patients suffering from breast cancer [34]. Some studies have also been suggested that circRNAs can play a role as biomarkers of HCC [35–37]. However, there is a lack of clarity regarding the role of circRNA as a biomarker of HCC.

In this study, sequencing technology was used for the evaluation of differentially expressed circRNAs. Post-screening and sorting, the four highly up-regulated circRNAs (hsa_circ_0008444, hsa_circ_0006091, hsa_circ_0049914, and hsa_circ_0091561) were identified. These circRNAs may serve as candidate biomarkers for HCC. Further verification was carried out via RT-PCR and qPCR, and the obtained results revealed that hsa_circ_0006091 showed considerable differential expression in HCC and adjacent tissues. In this study, the underlined circRNA was reported in HCC for the first time. In this study, we used bioinformatics tools for extensively studying the miRNA and mRNA networks and signal cascades associated with hsa_circ_0006091 in HCC. The regulatory network of hsa_circ_0006091 contains 4 miRNAs and 230 target genes. The pathway analysis and functional analysis demonstrated that hsa_circ_0006091 significantly contributes to the occurrence and development of liver carcinoma.

Based on the ceRNA network theory, the expression level of circRNA is positively correlated with its target genes. GO and KEGG analyses were used for the evaluation of various genes, such as QKI, EGFR, and RGS12 that are closely associated with the incidence of cancer. Thus, we speculate that these genes may be differentially expressed in HCC tissues and can serve as a marker of HCC. Hence, these genes were identified and confirmed. The obtained results indicated the elevated expression level of RGS12 in HCC tissues. We conducted a ROC curve diagnosis on hsa_circ_0006091 and RGS12, accordingly. Simultaneously, the combined diagnosis was carried out. The obtained results revealed that the combined diagnosis has higher sensitivity than that of the individual diagnosis. These results indicate that hsa_circ_0006091 and its parent gene RGS12 can be used as diagnostic markers for HCC.

The current study has some limitations. First, the number of verified samples is comparatively small, particularly the number of samples in the blood which can lead to selection bias. For further verification, a larger number of samples will be required. Second, the current research emphasizes expression and diagnosis. The extensive molecular mechanism of hsa_circ_0006091 in HCC has not been explored. Third, it is needed to identify the expression level of hsa_circ_0006091 in other tumors to see whether it is specific in HCC or contributes to other tumors as well. In a future study, we will emphasize it.

## 5. Conclusion

In summary, this study has been revealed that hsa_circ_0006091 and RGS12 have an elevated expression level in HCC tissues. The joint diagnosis of the two has diagnostic significance and can be used as a molecular marker for HCC diagnosis. Meanwhile, hsa_circ_0006091 combined with AFP was more sensitive and specific in the diagnosis of HCC.

## Supplementary Material

Supplemental Material

## Data Availability

For original data, please contact yebenqian126@126.com. Detailed data may be found in ‘Supplemental Table 1-5’.

## Research highlights

- Hsa_circ_0006091 was highly expressed in HCC tissues and serum.
- RGS12 was highly expressed in HCC tissues compared with paracancer tissues.
- Hsa_circ_0006091 and RGS12 have certain diagnostic values for HCC.
- Hsa_circ_0006091 combined with AFP or RGS12 can improve the efficiency of HCC diagnosis.

## Research in context

Evidence before this study

The incidence and mortality of liver cancer have remained high in recent years. Early diagnosis is the key to the treatment and prognosis of HCC patients. Although AFP is a clinical diagnostic marker for HCC, however, its sensitivity and specificity are not ideal. Therefore, new HCC biomarkers need to be discovered. Circular RNA will be the focus of our research as a new HCC biomarker.

Added value of this study

This study found that hsa_circ_0006091 is highly expressed in liver cancer tissues, with a significant difference when compared to adjacent tissues. In addition, dynamic monitoring of patient’s serum before and after surgery found that the hsa_circ_0006091 level was significantly higher in HCC patients before surgery and tumor recurrence than after surgery and without recurrence. Bioinformatics analysis found RGS12 to be highly expressed in liver cancer tissues.

Implications of this study

This study found that hsa_circ_0006091 has a high predictive value for HCC patients and that its combination with AFP/RGS12 can increase the diagnostic efficiency of HCC patients.
