# Supplementary material for: Circular RNA hsa_circ_0006091 as a novel biomarker for hepatocellular carcinoma
Source: Bioengineered. 2022 Feb 3;13(2):1988–2003. doi: 10.1080/21655979.2021.2006952 (PMC8973770; doi:10.1080/21655979.2021.2006952)
Supplement: Supplemental Material [file KBIE_A_2006952_SM0973.zip › supplementary/Table S5.docx]

Table S5 : The four miRNA targets predicted by MiRWalk and TargetScan database.

| hsa-miR-1286 |
| --- |
| PCDH17 GAS7 KIF21B NFAT5 SOGA1 LUC7L3 HMGN2 MMP16 RHOA HNRNPK MEF2C CD47 ASPH DYRK1A ZMYND11 DLG2 ZNF423 SH2D4B FNBP1L LARP1 FBXO11 IPCEF1 RORB PLEKHB2 QKI BACH2 NAA50 FOXP1 USP42 JADE2 HIF1AN CNOT2 AGO1 SLC39A10 TOGARAM1 KLF12 MAFB |
| hsa-miR-1248 |
| PTPDC1 TRIM24 LDLRAD4 RAD51B PRKAB2 KALRN KPNA6 TBL1X SRSF10 MYCBP2 EIF3J TPM3 RARB DMD PAX3 SMARCA1 RBMS1 CCND2 BMI1 PAFAH1B1 MAP2K4 RFX3 NOVA1 CLCN3 NXPH1 CD83 TLE4 CD164 MBTD1 ELAVL2 TIAM1 CDC16 MTAP ADAM9 PICALM RUNX2 DPYSL3 GAPVD1 SUZ12 NONO DLG2 NREP HIF1A RFX7 NIPBL CDC73 NHS PRICKLE2 KTN1 SESTD1 CHSY1 PDSS2 NAV3 DTWD2 UHMK1 RB1CC1 SH3TC2 PDCD6IP SLC7A6 BCL2L2 FBXW7 DGAT2 QKI DLC1 HBEGF KLF6 PIP5K1A ZYG11B RNF38 AGO4 BCLAF1 TCF20 MED13 DNM3 RIMS2 NTNG1 RNF11 DTNA CSNK1G3 |
| hsa-miR-1197 |
| DRGX HRK FOXN3 LZIC SPOCK2 ZNF592 NEO1 CELF1 GLG1 COPS5 EXTL1 TNPO1 FBXO32 UTP4 SLC25A36 ZNF501 RNF125 MARS2 FUBP3 MFSD14A QKI SCN2A HNRNPAB TMEM47 UPF3B NMNAT2 XPO4 GZF1 KLK15 ACBD3 HDAC5 HS3ST2 SLC35A3 ICE1 POLR3K KLF13 UBE2J1 KIF3A RAPGEF2 PAXBP1 SELENOK |
| hsa-miR-1231 |
| AIF1L AMPH ANKRD13B ARL5A ATG5 AZIN1 BTG1 BTN3A2 CCP110 CHD7 CLEC16A CNOT6 CREB3L3 CREBL2 CTDSPL DCX DDX17 DEDD DNAJC11 DPP3 EIF4B ELMSAN1 EVI5 FGFR1OP GLG1 GLP1R GPAM HDGF HDGFL3 HECTD4 IGF1 IPO9 JAZF1 KCNA1 KDM5A KPNA1 KPNA6 LARP4B LMX1B MAP1A MAP2K4 MECP2 MEIS2 METTL9 MYH10 NAA15 NAMPT NBPF14 NCOA2 NEFM NOTCH2 NOVA1 NR2C2 NTNG1 NTRK2 ONECUT2 PACSIN1 PAFAH1B1 PIK3R1 PNN PPP1R9A PPP2R1B PRDM16 PRKCI PSIP1 QKI ZFX ZIC1 ZNF215 ZNF592 |
