# Supplementary material for: Circular RNA hsa_circ_0006091 as a novel biomarker for hepatocellular carcinoma
Source: Bioengineered. 2022 Feb 3;13(2):1988–2003. doi: 10.1080/21655979.2021.2006952 (PMC8973770; doi:10.1080/21655979.2021.2006952)
Supplement: Supplemental Material [file KBIE_A_2006952_SM0973.zip › supplementary/TableS3.docx]

| CircRNA ID | Gene symbol | Base Mean | log2  Fold Change | P value | padj | Regulation |
| --- | --- | --- | --- | --- | --- | --- |
| hsa_circ_0036682 | ABHD2 | 96.81584511 | -10.28373199 | 7.38E-06 | 0.002531672 | Down |
| hsa_circ_0026921 | SLC39A5 | 64.58384039 | -9.627948524 | 5.33E-05 | 0.008708948 | Down |
| hsa_circ_0027020 | GLS2 | 60.30415067 | -9.463711623 | 9.12E-05 | 0.010431249 | Down |
| hsa_circ_0025580 | SLCO1B3 | 45.40300059 | -9.053717877 | 0.000238374 | 0.016736186 | Down |
| hsa_circ_0089900 | TC EANC | 46.97973725 | -9.050474348 | 0.000260113 | 0.016736186 | Down |
| hsa_circ_0057714 | AOX1 | 42.83418981 | -8.951239055 | 0.000308313 | 0.018662018 | Down |
| hsa_circ_0019246 | CYP2C19 | 39.58834966 | -8.814066654 | 0.000427269 | 0.023581631 | Down |
| hsa_circ_0068513 | TPRG1 | 40.80479977 | -8.811014334 | 0.000458341 | 0.023581631 | Down |
| hsa_circ_0045468 | BPTF | 39.8201355 | -8.798809771 | 0.000455751 | 0.023581631 | Down |
| hsa_circ_0012745 | FGGY | 37.41915502 | -8.719120403 | 0.000530749 | 0.024824601 | Down |
| hsa_circ_0054943 | UGP2 | 35.19769488 | -8.587497143 | 0.000732437 | 0.029381851 | Down |
| hsa_circ_0069718 | DCUN1D4 | 33.93208082 | -8.563800638 | 0.000742399 | 0.029381851 | Down |
| hsa_circ_0066852 | ZBTB20 | 32.74755045 | -8.519165522 | 0.000803766 | 0.030632419 | Down |
| hsa_circ_0084195 | SPIDR | 32.66053375 | -8.491957656 | 0.000871423 | 0.03179604 | Down |
| hsa_circ_0076149 | FKBP5 | 31.71830062 | -8.46686198 | 0.000897935 | 0.03179604 | Down |
| hsa_circ_0020237 | FGFR2 | 31.2712888 | -8.432821973 | 0.000973786 | 0.03179604 | Down |
| hsa_circ_0005805 | SLC7A2 | 30.92083662 | -8.421991888 | 0.000988798 | 0.03179604 | Down |
| hsa_circ_0079705 | LOC401320 | 29.41292533 | -8.333454756 | 0.001193796 | 0.03722472 | Down |
| hsa_circ_0003731 | PTGR1 | 169.1244001 | -6.025943244 | 0.000181644 | 0.016672573 | Down |
| hsa_circ_0006091 | RGS12 | 39.96218081 | 8.67386629 | 0.000613286 | 0.026294633 | Ups |
| hsa_circ_0049914 | MYO9B | 54.04104524 | 9.135581966 | 0.000217995 | 0.016736186 | Ups |
| hsa_circ_0008444 | SDHAP2 | 52.31973947 | 9.145281673 | 0.000194432 | 0.016672573 | Ups |
| hsa_circ_0091561 | LOC286467 | 60.51972391 | 9.320541296 | 0.000137822 | 0.014181864 | Ups |

TableS3: High-throughput sequencing analysis of differential circular RNAs.
