# Supplementary material for: Circular RNA hsa_circ_0006091 as a novel biomarker for hepatocellular carcinoma
Source: Bioengineered. 2022 Feb 3;13(2):1988–2003. doi: 10.1080/21655979.2021.2006952 (PMC8973770; doi:10.1080/21655979.2021.2006952)
Supplement: Supplemental Material [file KBIE_A_2006952_SM0973.zip › supplementary/TableS4.docx]

TableS4: The information of circular RNA and [target](javascript:;) [gene](javascript:;) primers.

| Amplicon | Primers | Gene symbol | Product length |
| --- | --- | --- | --- |
| hsa_circ_0049914 | F:5’- AGCCAGTGAAGCAGAGCAA-3′  R:5’- CCTGCCTCTTTCACACTCAT-3′ | [MYO9B](http://www.ncbi.nlm.nih.gov/gene/?term=4650) | 131 bp |
| hsa_circ_0008267 | F:5’- AAGTGGTTGCTGTGGAGGAC-3′  R:5’- TGATGTGTTGTCCTCTGGCT-3′ | [SDHAP2](http://www.ncbi.nlm.nih.gov/gene/?term=727956) | 87 bp |
| hsa_circ_0001944 | F:5’- AGGGCATGGATCACTAAGGT-3′  R:5’- GCAGAGCAGCTTGAAGAACA-3′ | TCONS 12 | 193 bp |
| hsa_circ_0006091 -1 | F:5’- TCGCTCCCTTGATGATCTTGA-3′  R:5’- AGCACGTCTCATTGTTCCCT-3′ | RGS12 | 100 bp |
| hsa_circ_0008444 | F:5’- TGCAGCTGACAGAAACTCATG-3′  R:5’- TGTGTTGTCCTCTGGCTGTG-3′ | SDHAP2 | 137 bp |
| Hsa-circ-0091561 | F:5’- TGTTCTTCAAGCTGCTCTGC-3′  R:5’- TCAGGTCTCACATCACATAAGTC-3′ | LOC286467 | 190 bp |
| β-actin | F: 5’-GGGAAATCGTGCGTGACATTAAG-3′  R:5’- TGTGTTGGCGTACAGGTCTTTG-3′ | β-actin | 240 bp |
| hsa_circ_0006091 -2 | F:5’- CTCAAAATTTGGGCGGGGAA-3′  R:5’- ACAGTGCCCTCAAGATCATCA-3′ | RGS12 | 123 bp |
| CTNNA1 | F:5’- CCATTTCCTCCTCCTAGCCG-3′  R:5’-GTTGTAACCTGTGTAACAAGAGGC3′ | CTNNA1 | 208 bp |
| GSK3B | F:5’- GGCACATCCTTGGACTAAGGTC-3′  R:5’- TGCTTGAATCCGAGCATGAGGA-3′ | GSK3B | 186 bp |
| RGS12 | F:5’- AGTCAGAAAGGACAGTGGGC-3′  R:5’- CAAGAGCCTCTCAGGACACC-3′ | RGS12 | 194 bp |
| QKI | F:5’- GTGTATTAGGTGCGGTGGCT-3′  R:5’- ATAGGTTAGTTGCCGGTGGC-3′ | QKI | 202 bp |
|  |  |  |  |
